# The synthesis, secretion and uptake of prorenin in human amnion

**DOI:** 10.14814/phy2.12313

**Published:** 2015-04-22

**Authors:** Kirsty G Pringle, Yu Wang, Eugenie R Lumbers

**Affiliations:** Mothers and Babies Research Centre, Hunter Medical Research Institute, John Hunter Hospital & School of Biomedical Sciences and Pharmacy, University of NewcastleNewcastle, New South Wales, Australia

**Keywords:** Amnion, amniotic fluid, human pregnancy, pregnancy outcome, prorenin

## Abstract

Very high concentrations of prorenin protein occur in human amniotic fluid and amnion. The source of amniotic fluid prorenin is likely the decidua, as it has the highest levels of prorenin mRNA (*REN*). Conversely, *REN* mRNA levels in amnion and chorion are very low. This study aimed to investigate whether decidual prorenin could cross the amnion and accumulate in amniotic fluid. Late gestation amnion was incubated for 24 h in the presence or absence of recombinant human (rh)prorenin. *REN* mRNA abundance was determined by qPCR and prorenin protein levels in the supernatant and tissue were measured by an ELISA. Prior to incubation only 3/11 amnion samples had *REN* mRNA but measurable levels of prorenin protein were found (1.4 ng/mg total protein). After 24 h incubation, *REN* mRNA was found in all explants and levels were significantly increased (*P *=* *0.03) but prorenin protein levels in amnion were unchanged. Prorenin protein levels in the supernatant were, however, increased (*P *=* *0.048). Incubation with (rh)prorenin significantly increased amnion tissue prorenin levels (2.8 ng/mg total protein, *P *=* *0.001); *REN* mRNA levels were unchanged. Therefore, amnion explants express small amounts of *REN* and secrete prorenin protein. Prorenin is also taken up by amnion. We postulate that the amniotic renin angiotensin system (RAS) alters pregnancy outcome through effects on gestation length and amniotic fluid volume. Since human amnion can take up and secrete prorenin protein, the activity of both amnion and amniotic fluid RASs can be amplified by prorenin produced by other intrauterine tissues.

## Introduction

Prorenin is a big molecule (46 KDa). It is the inactive precursor of renin, the rate-limiting enzyme in the renin-angiotensin system (RAS) cascade, which primarily acts to control blood pressure and salt and water homeostasis. The active enzyme renin is a specific aspartyl protease responsible for cleaving the decapeptide, angiotensin (Ang) I from liver derived angiotensinogen (AGT) (Reudelhuber et al. 1994; Neves et al. 1996). A prorenin receptor, identified by Nguyen et al. 2002, nonproteolytically activates prorenin, so prorenin can activate the RAS or function independently of the RAS, via intracellular signaling (Nguyen et al. 2002).

As well as the circulating RAS there are various organ and tissue-based RASs, including those found in human intrauterine tissues (Marques et al. 2011; Pringle et al. 2011a,b). These may act as paracrine or autocrine systems. They are different from the circulating RAS as they only secrete prorenin (Pringle et al. 2011a). The kidney is the only organ that can secrete active renin.

In late gestation human pregnancy, the human decidua (maternal lining of the uterus) is the major site of prorenin production (Shaw et al. 1989; Pringle et al. 2011b). Next to the kidney, the highest levels of prorenin are found in human amniotic fluid (Skinner et al. 1968; Itskovitz et al. 1992), yet as we have found, mRNA expression of prorenin in amnion and chorion is very low (Pringle et al. 2011b). If the decidua is the major site of production of prorenin in intrauterine tissues and it is this prorenin that is secreted into amniotic fluid, it follows that decidual prorenin has to cross the amnion, an epithelial cell barrier. This would involve uptake and release of prorenin by amnion epithelial cells.

Since prorenin is a large molecule, its uptake by cells requires a receptor. Two receptors are primarily responsible for uptake of prorenin, the insulin-like growth factor II receptor (IGF2R, also known as the mannose-6 phosphate receptor), and the prorenin receptor (Batenburg and Danser 2012). Since human amniotic fluid renin is not mannosylated, it is unlikely that the IGF2R is involved in the uptake of decidual prorenin by chorion or amnion, if it is the source of amniotic fluid prorenin. We have, however, shown that the prorenin receptor is abundantly expressed in human amnion chorion and decidua (Pringle et al. 2011b). The (P)RR is associated with vacuolar H(+)-ATPase (V-ATPase), a multi-subunit proton pump involved receptor-mediated endocytosis and recycling, processing of proteins and signaling molecules, membrane sorting and trafficking, and activation of lysosomal/autophagosomal enzymes (Kinouchi et al. 2010). Therefore, the prorenin receptor may be involved in taking up prorenin from the decidua.

Although prorenin in amniotic fluid is generally considered inactive, about 1% of the very high levels of amniotic fluid renin, present mainly as prorenin, is spontaneously converted into active renin (Derkx et al. 1987). Additionally, prorenin binding to the prorenin receptor on the amnion cell membrane or to the soluble form of the prorenin receptor (Senbonmatsu et al. 2010) in amniotic fluid could have both angiotensin-dependent and angiotensin-independent actions that might profoundly affect pregnancy outcome (Nguyen et al. 2002). Recently, we have proposed a mechanism by which the prorenin/prorenin receptor interaction could act to maintain the integrity of the fetal membranes (Pringle et al. 2014).

To determine whether decidual prorenin could cross these membranes and accumulate in amniotic fluid, we tested the hypotheses that (1) human amnion constitutively secretes small amounts of prorenin protein; and (2) prorenin from an exogenous source (such as the decidua) can be taken up and stored in human amnion explants incubated *in vitro*.

## Methods

### Tissues

Amnion was collected from uncomplicated singleton pregnancies at term (37–41 weeks gestation), delivered by elective cesarean section in the absence of labor (nonlaboring, *N* = 11). Women treated with nonsteroidal anti-inflammatory drugs, or having a history of infection, chorioamnionitis or asthma, or undergoing labor induction were excluded. Informed consent was obtained from all participants, as approved by the Hunter Area Research Ethics Committee and the University of Newcastle Human Research Ethics Committee.

For immunohistochemical analysis, strips of full thickness membranes (amnion, chorion and decidua), dissected away from the adjacent placenta and cervix, were fixed in 4% paraformaldehyde overnight, before embedding in paraffin.

### Amnion incubation

Amnion membrane was cut into approximately 2 cm^2^ pieces and distributed into 0.5 g portions. Each portion was then washed briefly in sterile phosphate-buffered saline (PBS) and incubated in 25 mL DMEM/F-12 medium (DMEM/F-12 supplemented with 15 mmol/L HEPES 1.2 g/L NaHCO_3_, 1 mg/mL L-glutathione reduced, 0.1 g/L Albumin Fraction V, 0.65 μg/mL Aprotinin and 40 μg/mL gentamicin) with 0 or 50 ng/mL recombinant human (rh) prorenin (Cayman Chemicals). Incubations were performed for 0 or 24 h at 37°C. After incubation, the tissues were removed from the medium, blotted and frozen in liquid nitrogen until extraction of RNA or protein. Zero hour samples were frozen immediately without incubation.

### Quantitative real-time reverse transcription-coupled polymerase chain reaction (qRT-PCR)

Total RNA was extracted using TRIzol reagent (Invitrogen, Carlsbad, CA) according to the manufacturer's instructions. RNA samples were DNAse treated on spin columns (Qiagen N.V., Hilden, Germany) to eliminate contaminating genomic DNA. Total RNA from incubated amnion samples were spiked with Alien RNA (Stratagene, La Jolla, CA; 10^7^ copies per microgram of total RNA), which served as reference RNA for internal standardization (Gilsbach et al. 2006). RNA was reverse transcribed using the SuperScript III RT-kit with random hexamers (Invitrogen). Quantitative real-time PCR (qPCR) was performed in an Applied Biosystems 7500 Real Time PCR System using SYBR Green for detection. Each reaction contained 5 μL SYBR Green, primers, cDNA and water to 10 μL. The primer pairs were designed using Primer Express software with one primer spanning an exon–exon junction. The following primers were used to detect *REN* mRNA expression: Forward 5′-CCACCTCCTCCGTGATCCT – 3′; Reverse 5′- GCGGATAGTACTGGGTGTCCAT – 3′. Where a sample failed to amplify after 40 cycles (i.e., was undetectable), it was given a CT value of 41. Messenger RNA abundance was calculated relative Alien mRNA using the ΔC_T_ method. Comparisons of mRNA abundance between PCR runs were made by incorporating a calibrator sample in each set of PCR amplifications and determining relative abundance as 2^−ΔΔCT^ (Livak and Schmittgen 2001). Dissociation curves, to check for homogeneity of amplification products, were generated for all reactions, and no-template control samples were included in all assays to confirm the absence of nonspecific amplification products due to primer interactions. The predicted sizes of the PCR products were verified by agarose gel electrophoresis (data not shown).

### Protein extraction

Amnion tissue was snap frozen and crushed in liquid nitrogen. For each sample, 0.2 g crushed amnion tissue was homogenized on ice in 2 mL protein extraction buffer containing 50 mmol/L Tris HCl, 150 mmol/L NaCl, 5 mmol/L MgCl_2_, 1 mmol/L EDTA, 1% Nonidet-P40, 0.5% sodium deoxycholate, 1× Complete Mini protease inhibitor cocktail (Roche, Basel, Switzerland), 1× PhosSTOP (Roche) and 1 mmol/L PMSF. Following homogenization samples were incubated on ice for 30 min and centrifuged at 17,000 *g* at 4°C. Supernatant containing the extracted protein (nuclear and cytosolic) was collected and quantified using a BCA Assay (Thermo Scientific, Rockford, IL).

### Prorenin ELISA

Prorenin levels in the supernatant and amnion tissue were measured by a human prorenin ELISA (Molecular Innovations Inc, Novi, MI) according to the manufacturer's instructions as previously described (Wang et al. 2012). Samples were assayed in duplicate. Since all samples were assayed on one ELISA plate there was no inter-assay variability. Intra-assay coefficient of variation was 3.65%. In our laboratory, 1 ng/mL human amniotic fluid prorenin measured using this ELISA could, when acid-activated, generate 116 ng h^−1^ mL^−1^ of Ang I at pH 7.5 and 37°C from angiotensinogen present in nephrectomized sheep plasma.

### Immunohistochemistry

Four μm thick formalin-fixed paraffin embedded sections were deparaffinized, and antigen retrieval was performed using a microwave oven with Novocasta™ Epitope Retrieval Solution (Leica Biosystems, Newcastle upon Tyne, UK) for 10 min. Sections were blocked with 2% skim milk powder in Tris-buffered saline (TBS) and then incubated for 1 h with primary antibody (anti-human renin propeptide; MAB4447; R&D Systems, Minneapolis, MN). Normal human kidney tissue was used as a positive control and matched samples without the use of the primary antibody were used as negative controls. Immunostaining was performed on a Bond-X™ automated immunostainer (Leica MicroSystems, Wetzlar, Hesse, Germany) with the Bond Polymer Refine Detection System (Leica MicroSystems) consisting of polymer conjugated anti-mouse/rabbit secondary antibody and diaminobenzidine (DAB, brown) as the chromogen. Images were captured and analyzed using the Aperio Scanscope XT slide scanner (Leica MicroSystems).

### Data analyses

Paired *t*-tests were undertaken to determine the effect of incubation time and prorenin treatment in amnion explants. GraphPad® Prism 6 (La Jolla, CA) was used for the analyses. Significance was set at *P *<* *0.05.

## Results

### Human amnion can express *REN* mRNA and secrete small amounts of prorenin *in vitro*

**Figure 1:**
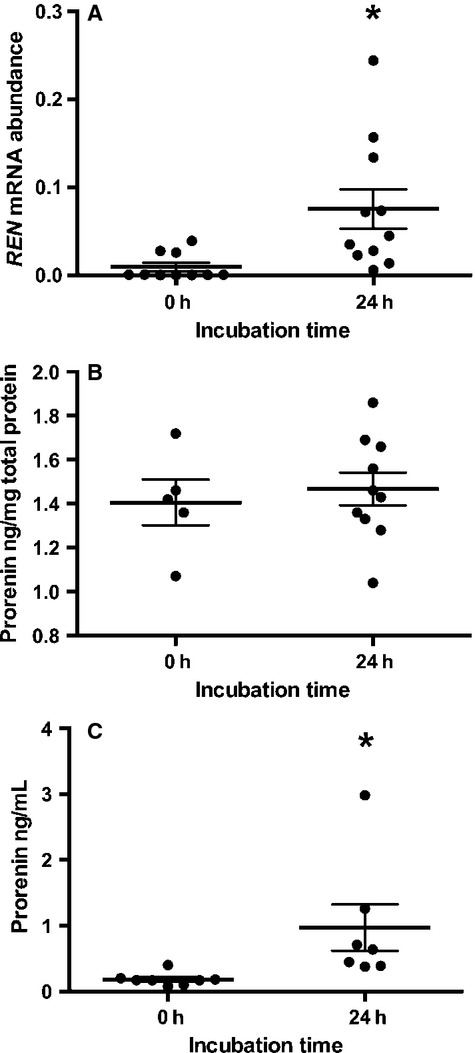
Prorenin mRNA and protein levels in incubated amnion and supernatant after 0 and 24 h incubation. (A) prorenin mRNA levels were significantly increased after 24 h incubation. (B) Prorenin protein levels in amnion tissue extracts remained the same, while protein levels in the supernatant were significantly increased (C). Data are presented as mean ± SEM. *denotes significant difference from 0 h time (*P *<* *0.05). *N* = 5–11 amnion per group.

At 0 h (prior to incubation) only 3/11 amnion had detectable levels of *REN* mRNA (Fig.1A). After 24 h, all 11 amnion explants had detectable levels of *REN* mRNA abundance and the expression of *REN* mRNA was significantly increased (*P *=* *0.028).

At 0 h amnion tissue had significant levels of prorenin protein (Fig.1B) despite the negligible expression of *REN* mRNA; there were only low levels of prorenin protein in the supernatant at this time (Fig.1C). Prorenin levels in amnion tissue did not change with incubation for 24 h (Fig.1B), but prorenin levels in the supernatant were significantly increased (*P *=* *0.048; Fig.1C).

**Figure 2:**
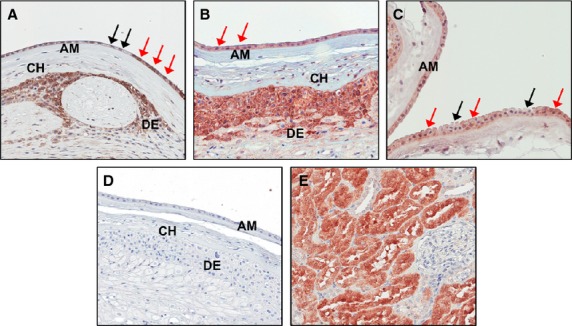
Immunohistochemical localization of prorenin in freshly isolated term full thickness membranes. (A–C) Despite the very low level of expression of *REN* mRNA in amnion, prorenin protein was found in the cytoplasm of amnion epithelial cells; images from three different samples are shown. Many cells displayed strong staining for prorenin (red arrows) while others had little to no staining (black arrows). Immunostaining was not seen in the negative control (D) containing no primary antibody. Normal human kidney was used as a positive control (E). Unlike active renin which is specifically localized to the juxtaglomerular cells of the kidney, prorenin could be detected in renal tubules. Images were photographed at 20× magnification. AM, amnion; CH: chorion; DE, decidua.

Figure2 shows that abundant prorenin protein is localized to the cytoplasm of amnion epithelial cells from freshly isolated amnion. Importantly, the distribution of prorenin across amnion epithelial cells is not homogenous in many samples. Some cells have weak or no staining for prorenin while others are densely stained.

### Amnion epithelial cells incubated *in vitro* can take up exogenous (rh)prorenin

**Figure 3:**
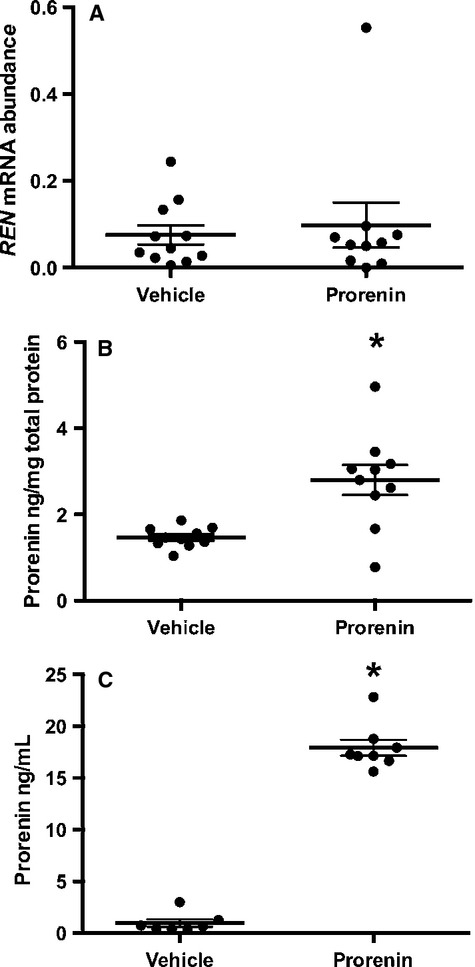
Prorenin mRNA and protein levels in amnion and supernatant after incubation for 24 h with 0 or 50 ng/mL (rh)prorenin. (A) *REN* mRNA levels were not altered by treatment with (rh)prorenin. (B) After 24 h incubation with (rh)prorenin, incubated amnion tissue contained significantly more prorenin than vehicle-treated controls there was also a significant increase in prorenin levels in the supernatant (C). Data are presented as mean ± SEM. *denotes significant difference from vehicle-treated controls (*P *<* *0.05). *N* = 9–11 amnion per group.

After 24 h incubation with (rh)prorenin, incubated amnion tissue contained significantly more prorenin than vehicle treated controls (*P *<* *0.001, Fig.3B). There was no difference in *REN* mRNA abundance (Fig.3A). Because 50 ng/mL of (rh)prorenin was added to the incubation media there was also a significant increase in prorenin levels in the supernatant at 24 h (*P *<* *0.0001, Fig.3C).

## Discussion

Although freshly isolated amnion contains little or no *REN* mRNA, after 24 h *ex vivo* incubated amnion expressed low levels of *REN* mRNA and secreted only small amounts of prorenin into the supernatant. We do not know if this was the newly synthesized prorenin protein or secretion from the pool of prorenin protein (Fig.1B) that can be seen histologically (Fig.2). Nonetheless, amnion can secrete prorenin into amniotic fluid.

Human amniotic fluid from term pregnancies contains variable amounts of acid-activatable prorenin measured enzymatically and there appears to be continuous secretion of prorenin into human amniotic fluid because the concentration of prorenin in amniotic fluid draining to the exterior does not decrease over a 5.5 h period following artificial rupture of membranes (Lumbers 1970).

Does the secretion rate of prorenin protein explain the high levels of amniotic fluid prorenin, measured as activity of the enzyme following acid activation (Skinner et al. 1968)? We incubated 0.5 g of amnion in 25 mL of medium. The spontaneous secretion rate was 1 ng/mL or 25 ng of prorenin per incubate over 24 h. Therefore, 1 g of amnion has the capacity to secrete 50 ng of prorenin over 24 h. In our laboratory, a pooled sample of amniotic fluid prorenin was measured both by the ELISA and by an enzymatic method almost identical to that used by Skinner et al. (except for the source of nephrectomized sheep substrate). 1 ng of amniotic fluid prorenin protein measured by the ELISA generated 116 ng/mL/h of Ang I at pH 7.5 and 37°C. Therefore, 0.5 g of incubated amnion generated the equivalent (in terms of enzyme activity) of approximately 242 ng/g amnion/h of Ang I, which is a rate sufficient to explain the high amniotic fluid prorenin levels.

Amniotic fluid is largely produced by fetal urine and lung fluid but is also influenced by intramembranous exchange of fluid across the amnion into the fetal vessels (Beall et al. 2007; Brace et al. 2014). Fetal urine probably only contributes small amounts of prorenin to amniotic fluid, because the level of renin activity in human neonatal urine is very low (Kaulhausen et al. 1979). Maternal plasma is also unlikely to be the source of amniotic prorenin because total maternal plasma renin levels are only about 2.6% those of amniotic fluid (Skinner et al. 1968). It follows therefore that only secretion of amniotic prorenin could account for the levels measured in amniotic fluid.

Despite the lack of expression of *REN* in freshly isolated amnion, amnion contained, on average, 1.4 ng prorenin/mg cellular protein. This level did not change over 24 h, even though *REN* mRNA levels increased. Since there was no corresponding increase in cellular prorenin protein there may have been secretion of prorenin from the amnion cells equivalent to its production. We cannot determine if the secretion of prorenin by amnion explants was the secretion of newly synthesized enzyme or secretion of stored decidual prorenin.

The second question we addressed was whether or not the prorenin stored in amnion epithelial cells (Figs.1B and 2) could have been taken up from the adjacent decidua. To test this, we incubated amnion with medium containing 50 ng/mL of (rh)prorenin. Figure3B shows that amnion contained much more prorenin after 24 h incubation with (rh)prorenin; approximately 2.8 ng prorenin/mg cellular protein (Fig.3B). This is a 215% increase in amnion prorenin protein. Therefore, we can conclude that the amnion epithelial cells can take up prorenin from the extracellular fluid surrounding them, which explains the presence of immunohistologically detectable prorenin in freshly isolated amnion (Fig.2) and the presence of the protein in 0 h incubates (Fig.1B). Only about one-third of the total amount of (rh)prorenin added to the incubate was found in the medium after 24 h however, suggesting that there is, as well as secretion and uptake, removal of prorenin, perhaps by proteases released into the supernatant from the amnion tissue *in vitro*.

Immunohistological examination of human amnion shows that prorenin is located within the cytoplasm of some but not all amnion epithelial cells in many samples (Fig.2). Since amniotic fluid renin is not glycosylated (Morris 1978) and cellular uptake of proteins by the IGF2R depends on glycosylation of proteins (van Kesteren et al. 1997; Peters et al. 2002), it is reasonable to assume that the prorenin receptor, which we have identified as being in abundance in human amnion (Pringle et al. 2011a,b) is the major pathway via which prorenin accumulates. This pathway has also been identified in the human heart (Saris et al. 2006). The uneven distribution of prorenin in the amnion may be a reflection of the amount of prorenin that has been internalized by the prorenin receptor at any given time.

The role of the amniotic RAS and the RAS in amniotic fluid is unknown. There is evidence that these systems can influence the expression of a number of genes involved in maintaining the integrity of the amnion in late gestation, and also influencing uterine activity (Pringle et al., 2014). Ang II is a potent angiogenic factor that could stimulate vascularization of the chorion through its effects on expression of vascular endothelial growth factor (*VEGF*). Amniotic fluid volume is influenced by intramembranous exchange of fluid and solutes across the amnion into these fetal vessels (Beall et al. 2007). The amniotic prorenin/Ang II system may also influence amniotic fluid volume and composition, by altering sodium transport across the amnion. Amniotic fluid incubated on its own, *in vitro*, can generate Ang I (Skinner et al. 1968). Both Ang I and Ang II are present in amniotic fluid (Kaulhausen et al. 1979). Ang II alters sodium transport across epithelial cells in the kidney and gut (Wong and Johns 1996) and regulates the expression of the intestinal epithelial sodium-hydrogen exchanger (NHE3) via the Ang II type 1 receptor (AT_1_R) (Musch et al. 2009) which is also expressed in the human amnion (Marques et al. 2011).

In conclusion, the large amounts of prorenin in human amnion are due to the uptake of prorenin from the surrounding medium as the level of expression of *REN* is low. We propose that the source of this prorenin is the adjacent decidua. The rate of secretion of prorenin from the amnion is sufficient to sustain levels of prorenin found in amniotic fluid. By binding to the prorenin receptor in amnion prorenin can also be nonproteolytically activated and can stimulate both Ang II-dependent and independent pathways. These renin–angiotensin systems in the fetal membranes (amnion and chorion) and the amniotic fluid may be involved in maintaining the integrity of the fetal membranes and regulating intramembranous water and solute flux, possibly through the actions of Ang II on vascularisation of the fetal chorion or on sodium transport across the amnion.

## Conflict of Interest

The authors have nothing to declare.
